# Supplementary material for: Mutations in PCYT2 disrupt etherlipid biosynthesis and cause a complex hereditary spastic paraplegia
Source: Brain. 2019 Oct 22;142(11):3382–97. doi: 10.1093/brain/awz291 (PMC6821184; doi:10.1093/brain/awz291)
Supplement: awz291_Supplementary_Data [file awz291_supplementary_data.zip › awz291-Suppl_data/Supplementary_Data4.zip!/Supplementary information 3 - fibroblast lipidomics.zip!/Fibroblast lipidomics/Controle vs Patient1/Statistical plots and data/Heatmap sorted on VIP-score.pdf]

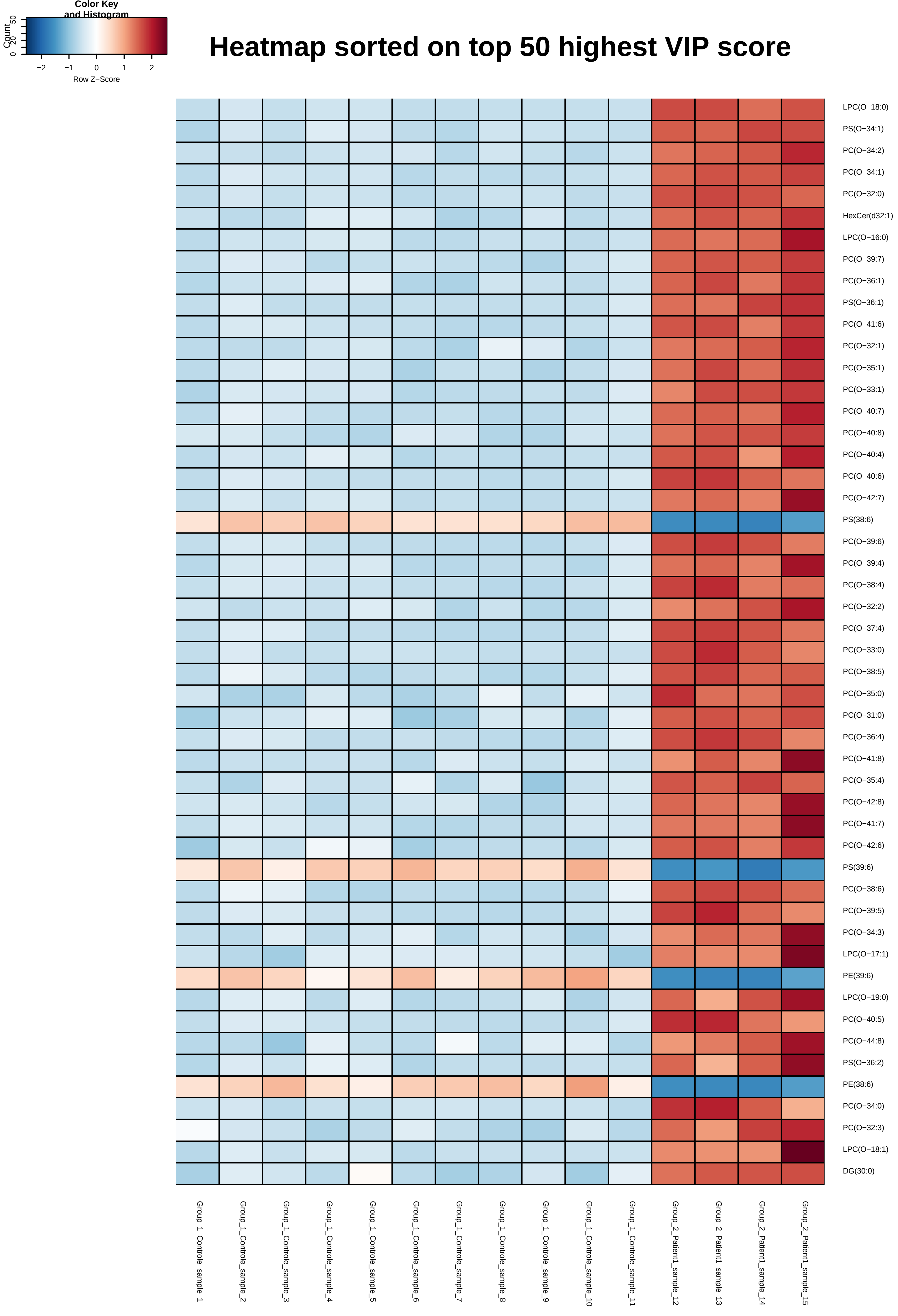
Color Key
and Histogram
Count
0
20
50
−2
−1
0
1
2
Row Z−Score
Heatmap sorted on top 50 highest VIP score
LPC(O−18:0)
PS(O−34:1)
PC(O−34:2)
PC(O−34:1)
PC(O−32:0)
HexCer(d32:1)
LPC(O−16:0)
PC(O−39:7)
PC(O−36:1)
PS(O−36:1)
PC(O−41:6)
PC(O−32:1)
PC(O−35:1)
PC(O−33:1)
PC(O−40:7)
PC(O−40:8)
PC(O−40:4)
PC(O−40:6)
PC(O−42:7)
PS(38:6)
PC(O−39:6)
PC(O−39:4)
PC(O−38:4)
PC(O−32:2)
PC(O−37:4)
PC(O−33:0)
PC(O−38:5)
PC(O−35:0)
PC(O−31:0)
PC(O−36:4)
PC(O−41:8)
PC(O−35:4)
PC(O−42:8)
PC(O−41:7)
PC(O−42:6)
PS(39:6)
PC(O−38:6)
PC(O−39:5)
PC(O−34:3)
LPC(O−17:1)
PE(39:6)
LPC(O−19:0)
PC(O−40:5)
PC(O−44:8)
PS(O−36:2)
PE(38:6)
PC(O−34:0)
PC(O−32:3)
LPC(O−18:1)
DG(30:0)
Group_1_Controle_sample_1
Group_1_Controle_sample_2
Group_1_Controle_sample_3
Group_1_Controle_sample_4
Group_1_Controle_sample_5
Group_1_Controle_sample_6
Group_1_Controle_sample_7
Group_1_Controle_sample_8
Group_1_Controle_sample_9
Group_1_Controle_sample_10
Group_1_Controle_sample_11
Group_2_Patient1_sample_12
Group_2_Patient1_sample_13
Group_2_Patient1_sample_14
Group_2_Patient1_sample_15
